# Assessment of knowledge and awareness regarding breast density among breast cancer screening healthcare professionals

**DOI:** 10.3389/fmed.2026.1843814

**Published:** 2026-07-09

**Authors:** Areej S. Aloufi

**Affiliations:** Department of Radiological Sciences, College of Applied Medical Sciences, King Saud University, Riyadh, Saudi Arabia

**Keywords:** breast cancer, breast density, breast imaging, health care professionals, mammography

## Abstract

**Background:**

Breast density (BD) is an established factor that reduces mammographic sensitivity and is associated with an increased risk of breast cancer. Healthcare professionals (HCPs) involved in breast cancer screening play a pivotal role in patient counseling, risk communication, and screening recommendations. This study aimed to assess knowledge and awareness regarding BD among this group of healthcare professionals in Saudi Arabia.

**Methods:**

A cross-sectional study was conducted using a self-administered online questionnaire distributed to the targeted HCPs in Saudi Arabia. Descriptive statistics were used to summarize participant characteristics and responses, and chi-square tests were used to examine associations between awareness variables and demographic characteristics, with statistical significance set at *p* < 0.05.

**Results:**

A total of 335 breast cancer screening HCPs participated in the study. Radiologists were more aware than non-radiology physicians and radiological technologists of the effect of BD on mammographic accuracy (87.6% vs. 70.0 and 76.7%, respectively) and breast cancer risk (83.5% vs. 58.0 and 65.6%, respectively). Awareness of the increased breast cancer risk associated with BD was significantly related to respondents’ specialty (*p* < 0.001) and years of professional experience (*p* < 0.001), while gender was significantly associated with awareness of reduced mammographic accuracy (*p* = 0.002).

**Conclusion:**

This study demonstrates variable awareness among breast cancer screening HCPs in Saudi Arabia regarding the impact of BD on mammographic accuracy and breast cancer risk, with significantly higher awareness among radiologists than among non-radiology physicians and radiologic technologists. In addition, the study highlights gaps in knowledge, patient communication, and routine clinical practices related to BD, emphasizing the need for targeted educational interventions.

## Background

Breast density (BD), an essential element in mammographic screening and evaluating breast cancer risk, has received growing focus in recent times (1). Increased BD is defined as the increased proportion of fibroglandular tissue in the breast (2). Studies have shown that women with higher BD have an increased likelihood of developing breast cancer compared to those with lower density (1–4). It is estimated that 10% of women have extremely dense breasts, doubling their risk of breast cancer (2). In Saudi Arabia, approximately 30% of women have increased BD, underscoring the need for heightened awareness and appropriate screening strategies in this population (5). Dense breast tissue can mask tumors on mammograms, making cancer detection more challenging and potentially delaying diagnosis (6).

Recent studies in Saudi Arabia have highlighted the association between increased BD and breast cancer risk, as well as its correlation with other risk factors (5, 7). Globally, several countries have recognized the importance of BD in breast cancer screening (1, 8, 9). In the United States, legislation mandates that women be informed about their BD and the potential need for additional screening beyond mammography (8). Similarly, the European Society of Breast Imaging (EUSOBI) recommends that women with extremely dense breasts be offered magnetic resonance imaging (MRI) screening every 2 to 4 years (1). These guidelines reflect a growing consensus on the need for personalized screening strategies based on BD. A study in 2019 surveyed 155 HCPs, including primary care providers, radiologists, and gynecologists, and found that more than two-thirds of the respondents (62%) were unaware of the increased risk of breast cancer associated with dense breasts. Additionally, two- thirds (67%) felt they needed more education about BD and supplemental screening, highlighting a gap in clinical practice (6). In the United Kingdom, 84% of radiologists and 100% of surgeons were aware of how BD affected mammogram accuracy and breast cancer risk in 2021. However, 52% of surgeons and 28% of radiologists believed that women should not be notified of their BD scores (4).

Whereas a study found that 97% of Australian general practitioners (GPs) had heard of BD, yet 87% expressed a need for more education, indicating a need for enhanced training on BD implications and management (10). In contrast, 48% of the physicians were unaware of BD laws, and 62% did not recognize the increased breast cancer risk associated with dense breasts. Additionally, 67% felt a need for more education on BD (6), while a systematic review among primary care practitioners (PCPs) exhibited a significant lack of knowledge about BD, low comfort in discussing it with patients, and limited consensus on managing women with dense breasts, particularly regarding supplemental screening approaches (11). In Saudi Arabia, there is a paucity of research on the awareness and attitudes of healthcare professionals regarding BD in the context of breast cancer screening, diagnosis, and treatment (12).

HCPs, specifically in breast cancer screening and imaging services, play a central role in educating patients about health conditions, available screening services, and preventive strategies, thereby influencing access to care and early detection practices (13, 14). Through routine clinical interactions and health-promotion activities, they shape individual decision-making as well as broader community health behaviors (15). In the context of breast imaging, their understanding of BD is particularly important, as it directly affects patient counseling, risk communication, and screening recommendations (13). Strengthening HCPs’ knowledge and attitudes toward BD is therefore essential to ensure accurate information delivery and informed shared decision-making (16), forming the basis for the present study on the assessment of HCPs’ knowledge and attitudes regarding BD. This proposed study aims to address this gap by conducting a multicenter exploratory study to evaluate the knowledge and awareness of breast cancer screening healthcare professionals (HCPs) in Saudi Arabia regarding BD.

## Methods

### Study design

A cross-sectional study was conducted over a one-year period from November 2024 to November 2025 to assess the awareness and knowledge towards BD. The target group included HCPs involved in breast cancer screening, diagnosis, or treatment within Saudi Arabia. For clarity, the term “HCPs” throughout this manuscript refers specifically to healthcare professionals working in breast cancer screening, breast imaging, diagnosis, or related breast cancer care, rather than the broader healthcare workforce. These providers represented hospitals, breast-imaging centers, oncology services, and primary care clinics engaged in mammography or breast-related care, or working in breast imaging or breast oncology services, and currently practicing in Saudi Arabia, who were directly involved in any aspect of breast cancer care and were willing to participate and provide informed consent. Those not practicing within Saudi Arabia, not involved in breast-related clinical services, or who submitted incomplete surveys were excluded. Before data collection, the study questionnaires were reviewed and approved by the central institutional review board at the Saudi Ministry of Health (approval under IRB No. 24-99E, dated 6/11/2024).

### Sample size and sampling technique

According to the 2023 Saudi Ministry of Health statistical report, more than 480,098 HCPs work in the country, including 113,300 physicians, 213,110 nurses, and 153,688 allied health personnel (17). Using a 95% confidence level and a 5% margin of error, the minimum required sample size was calculated using the standard formula for cross-sectional studies


n=(Z2×p×q)/E2

With Z = 1.96, p = 0.5, q = 1 − p, and E = 0.05, the calculated sample size was 384.16, rounded to 385 participants. A convenience sampling method was adopted to reach a large and diverse pool of HCPs across the country.

### Study questionnaire

Data were collected using an English-language survey adapted from a validated questionnaire developed by Varghese et al. (4). Permission was obtained from the original authors, who provided the complete instrument. To ensure suitability for the Saudi healthcare context, the adapted questionnaire was reviewed by a panel of nine experts, including seven radiological technologists and two radiologists with relevant experience in breast imaging and healthcare practice. The experts assessed the questionnaire for content relevance, clarity, readability, and appropriateness to the local clinical context.

Following expert review, the questionnaire was refined based on the feedback received. Pilot testing was then conducted among a small group of healthcare professionals to evaluate clarity, comprehension, wording, and ease of completion. Minor revisions were made before final implementation. The final questionnaire consisted of 19 items designed to assess healthcare professionals’ knowledge of breast density as a breast cancer risk factor, awareness of its effect on mammographic accuracy, reporting methods used in clinical practice, communication with patients, and perceptions of supplemental imaging for women with dense breasts (Supplementary File 1). Demographic information, including age, gender, specialty, current position, and years of professional experience, was also collected. The internal consistency of the modified questionnaire was assessed using Cronbach’s alpha. The overall scale demonstrated acceptable reliability, with a Cronbach’s alpha coefficient of ≥0.70, indicating satisfactory internal consistency for measuring healthcare professionals’ awareness and attitudes toward BD.

### Study population and recruitment

The questionnaire was administered online via the official survey platform of the Saudi Commission for Health Specialties (SCHS). It was distributed by the SCHS Research Support Department via email to eligible participants, and responses were collected anonymously. The survey invitation included information about the study’s purpose, procedures, confidentiality, and voluntary participation. The first page of the online survey included a detailed description of the study, and completion of the survey indicated informed consent.

### Data analysis

Categorical variables were summarized using frequencies (n) and percentages (%), while continuous variables were described using means and standard deviations. Chi-square tests were used to examine associations between breast density awareness variables and healthcare professionals’ demographic and professional characteristics. A *p*-value ≤0.05 was considered statistically significant.

In addition, multivariable binary logistic regression was performed to identify independent predictors of breast density awareness and selected clinical practices. The dependent variables were coded as binary outcomes, with “Yes” coded as 1 and all other responses, including “No,” “Unknown,” and “I don’t know,” coded as 0. The models were adjusted for age, gender, specialty group, and years of breast imaging experience. Female gender and non-radiology physicians were used as reference categories, and years of breast imaging experience was treated as an ordinal variable. Adjusted odds ratios (AORs), 95% confidence intervals (CIs), and *p*-values were reported. All analyses were performed using the Statistical Package for Social Sciences version 26.0 (SPSS Inc., Chicago, IL, USA).

## Results

**Figure 1:**
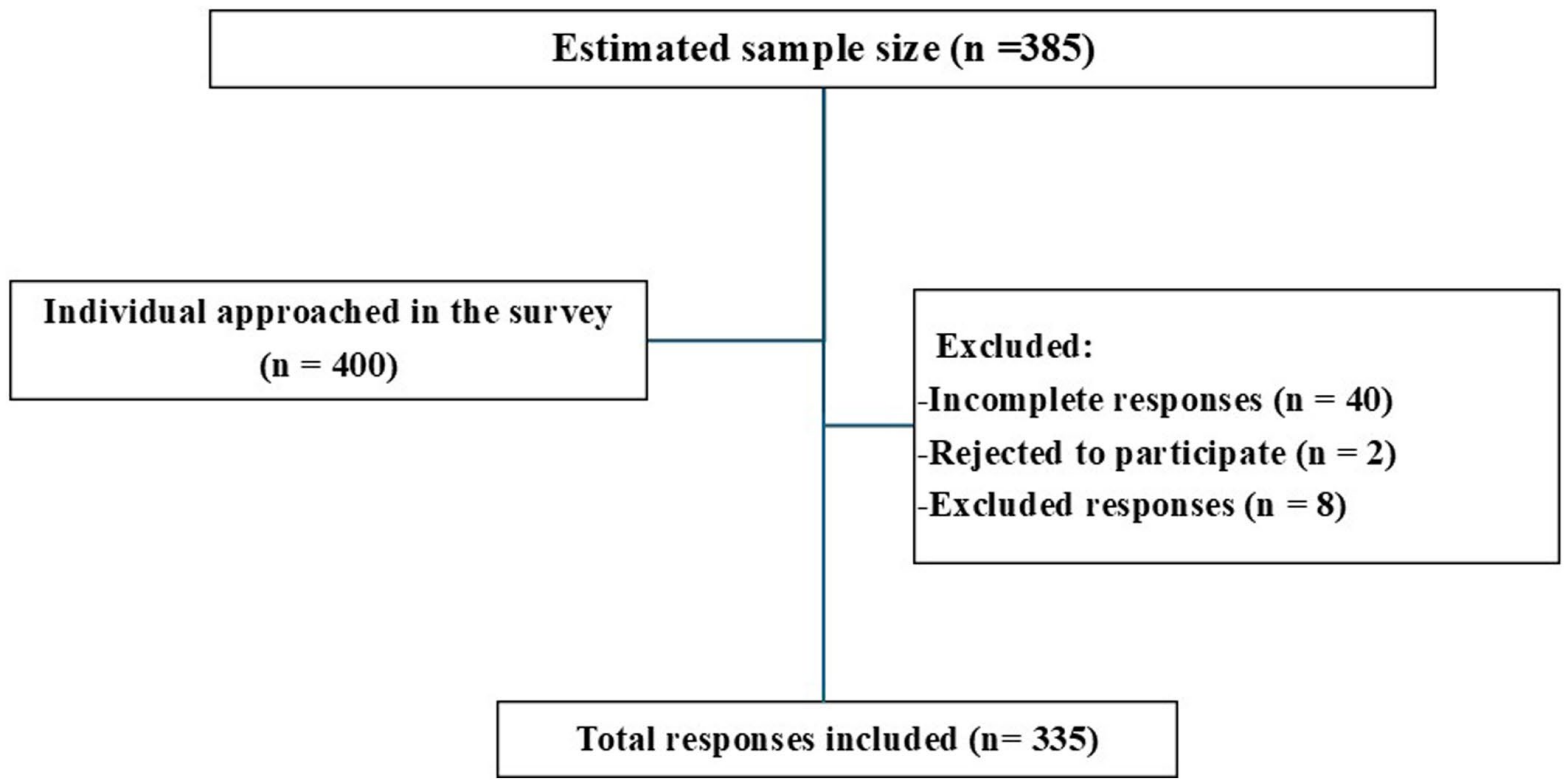
Flowchart of the responses.

A total of 335 HCPs (Figure 1), with a mean age of 39.8 years (SD ± 9.7), participated in this study; of these HCPs, 59.1% were female, while males accounted for 40.9% among physicians, consultants represented 30.5% of the sample, followed by registrars (27.5%). Eight responses were excluded from the final analysis because the participants had inactive healthcare practitioner licenses and were not currently working in a hospital in Saudi Arabia. Most HCPs who worked in breast imaging reported familiarity with breast ultrasound (67.8%) and mammography (61.2%). In contrast, fewer had experience with breast MRI (41.5%), digital breast tomosynthesis (26.3%), or contrast-enhanced mammography (17.3%). With respect to the duration of breast imaging experience, nearly one-third (30.4%) of HCPs reported one to 5 years of experience, and only 4.5% had more than 20 years of experience (Table 1).

**Table 1:** Characteristics of the study participants (*n* = 335).

Variables	Frequency (n)	Percentage (%)
Age (mean ±SD)	39.81 ± 9.7	
Gender		
Female	198	59.1%
Male	137	40.9%
Medical specialty group		
Radiologists	145	43.3%
Non-radiology physicians	100	29.9%
Radiological Technologists/mammographers	90	26.8%
Current position in the specialty		
Consultant	102	30.5%
Registrar	92	27.5%
Resident	21	6.2%
Fellow	20	5.9%
Radiological technologist	90	26.9%
Other	10	3%
Type of breast imaging experience, if any^*^		
Breast ultrasound	227	67.8%
Mammography	205	61.2%
Breast MRI	139	41.5%
Digital breast tomosynthesis (DBT)	88	26.3%
Contrast-enhanced mammography	58	17.3%
Years of breast imaging experience		
1–5 years (or less)	102	30.4%
5–10 years	60	17.9%
10–20 years	59	17.6%
>20 years	15	4.5%
None	99	29.6%

^*^Multiple choices question.

Awareness related to the knowledge of the effect of BD on mammographic accuracy was highest among radiologists, with 127 individuals (87.6%), compared with 70 (70.0%) among non-radiology physicians and 69 (76.7%) among radiological technologists. A similar trend was observed regarding awareness of the increased breast cancer risk associated with BD. In this case, 121 radiologists (83.5%) reported awareness, compared with 58 (58.0%) and 59 (65.6%) in the non-radiology physicians and medical technologists, respectively (Table 2).

**Table 2:** Awareness and reporting practices related to breast density.

Variables	Radiologists *n* = 145 *N* (%)	Non-radiology physicians *n* = 100 *N* (%)	Radiological technologists *n* = 90 *N* (%)	*p*-value
Are you aware that mammographic accuracy is affected by breast density?				
Yes	127 (87.6%)	70 (70.0%)	69 (76.7%)	<0.001
No	0 (0%)	17 (17.0%)	8 (8.9%)	
Unknown	18 (12.4%)	13 (10.0%)	13 (14.4%)	
Are you aware of the relative risk for breast cancer by the degree of breast density?				
Yes	121 (83.5%)	58 (58.0%)	59 (65.6%)	<0.001
No	6 (4.1%)	29 (29.0%)	18 (20.0%)	
Unknown	18 (12.4%)	13 (13.0%)	13 (14.4%)	
Does your department routinely report on mammographic density?				
Yes	107 (73.8%)	36 (36.0%)	42 (46.7%)	<0.001
No	12 (8.3%)	18 (18.0%)	12 (13.3%)	
I do not know	26 (17.9%)	46 (46.0%)	36 (40.0%)	
If yes, which ACR BI-RADS* category(s) are considered “dense breasts”				
Category A	4 (2.8%)	2 (2.0%)	5 (5.6%)	<0.001
Category B	2 (1.4%)	2 (2.0%)	6 (6.7%)	
Category C	10 (6.9%)	13 (13.0%)	22 (24.4%)	
Category D	16 (11%)	29 (29.0%)	12 (13.3%)	
Category C & D	104 (71.7%)	41 (41.0%)	33 (36.7%)	
Not applicable	9 (6.2%)	13 (13.0%)	12 (13.3%)	
Do you routinely offer supplementary imaging for women with increased breast density?				
Yes	91 (62.8%)	25 (25.0%)	27 (30%)	<0.001
No	14 (9.7%)	17 (17.0%)	12 (13.3%)	
Unknown	40 (27.6%)	58 (58.0%)	51 (56.7%)	
Are routine supplementary imaging results in over-investigation of false positive findings?				
Yes	38 (26.2%)	10 (10.0%)	14 (15.6%)	<0.001
No	52 (35.9%)	15 (15.0%)	13 (14.4%)	
I do not know	55 (37.9%)	75 (75.0%)	63 (70%)	

^*^According to ACR BI-RADS categories 5th edition (Category A: breasts almost entirely fatty, Category B: there are scattered areas of fibroglandular density, Category C: breasts are heterogeneously dense, and Category D: breasts are extremely denseand).

Knowledge of routine reporting of mammographic BD was more frequently seen among radiologists (73.8%). This practice was less seen by technologists (46.7%) and non-radiology physicians (36.0%). Notably, a considerable proportion of HCPs in the non-radiology physicians and radiological technologists reported uncertainty about whether BD was routinely included in reports. When asked about dense breast categories, most respondents correctly identified categories C and D as dense breasts. This was particularly evident among radiologists (104, 71.7%), compared with 41 (41.0%) in the non-radiology physicians and 33 (36.7%) in the radiological technologists group. The routine use of supplementary imaging for women with dense breasts was also most commonly reported by radiologists (91, 62.8%). In contrast, uncertainty regarding this practice was widespread among non-radiology physicians (58, 58.0%) and radiological technologists (51, 56.7%). Concerns about potential over-investigation were generally low across all groups; however, a high level of uncertainty persisted, particularly among non-radiology physicians (75, 75.0%) and radiological technologists (63, 70.0%).

In this study, 65.7% of HCPs agreed that patients should be informed about the reduced sensitivity of mammography associated with BD, and 51.6% believed that patients should also be informed about the increased risk of breast cancer. When asked about patient-initiated discussions, only 13.1% of respondents reported that patients ask about their BD, whereas 62.1% stated that they do not receive such inquiries. Practices related to sharing BD information varied among providers. Approximately one-third reported sharing this information only when patients specifically asked, 26.3% shared it routinely, and 21.8% reported that they did not share BD information at all. Among those who did not provide this information, the most commonly cited reasons included the belief that BD should not be discussed without supporting imaging, limited availability of information, low confidence in explaining the topic, and time constraints during consultations. In terms of patient guidance, providers most frequently recommended additional imaging (51.6%), encouraged breast awareness and self-examination (46.0%), or explained that BD can reduce the accuracy of mammography (42.7%). Only 18.5% specifically advised patients that higher BD is associated with an increased risk of breast cancer. Finally, more than half of the respondents (55.8%) indicated that national guidelines on BD are needed in Saudi Arabia (Table 3).

**Table 3:** Perceptions and patient awareness of breast density among HCPs.

Variables	Frequency (n)	Percentage (%)
Healthcare professionals should inform patients about reduced sensitivity.		
Yes	220	65.7%
No	32	9.6%
I do not know	83	24.8%
Healthcare professionals should inform patients about increased cancer risk.		
Yes	173	51.6%
No	79	23.6%
I do not know	83	24.8%
Do your patients ever ask about breast density?		
Yes	44	13.1%
No	210	62.7%
I do not know	81	24.2%
Do you share breast density information with your patients?		
Yes	88	26.3%
No	73	21.8%
Only if asked	112	33.4%
None	62	18.5%
Reasons for not sharing breast density information		
Do not feel this information should be shared without alternative imaging being offered	22	6.6%
Do not have this information available	15	4.5%
Do not feel confident sharing this info	11	3.3%
Time restraints	4	1.2%
Other	25	7.4%
Not applicable	258	77.0%
What advice regarding breast density do you offer your patients?^*^		
Breast awareness/breast examination	154	46.0%
Increased breast density may reduce the accuracy of a mammogram	143	42.7%
Increased breast density may increase breast cancer risk	62	18.5%
You may benefit from additional imaging due to your breast density	175	51.6%
Do you think there is a need for further guidelines in Saudi Arabia on the management of breast density in breast cancer screening and diagnosis?		
Yes	187	55.8%
No	67	20.0%
None	81	24.2%

^*^Multiple choices question.

Results showed that gender (*p* = 0.002) and specialty (*p* < 0.001) had a significant association with the awareness that mammographic accuracy is affected by BD. Also, specialty (*p* = <0.001) and year of experience (*p* = <0.001) had a significant association with the awareness of relative risk for breast cancer by the increased BD (Table 4).

**Table 4:** Association between demographic factors and the awareness of breast density effect on mammographic accuracy and cancer risk.

Demographic characteristics		Aware of the BD effect on mammography accuracy				Aware of BD and cancer risk			
		Yes	No	*I do not know*	*p*-value	Yes	No	*I do not know*	*p*-value
Gender									
Male	Count	106	18	13	0.002	97	27	13	0.102
	% within gender	31.60%	5.40%	3.90%		40.80%	50.90%	29.50%	
Female	Count	160	7	31		141	26	31	
	% within gender	47.80%	2.10%	9.30%		59.20%	49.10%	70.50%	
Specialty									
Non-radiology physicians	Count	70	17	13	<0.001	58	29	13	<0.001
	% within specialty	70.00%	17%	13%		58.00%	29.00%	13.00%	
Radiologists	Count	127	0	18		121	6	18	
	% within specialty	87.60%	0%	12.40%		83.50%	4.10%	12.40%	
Radiological Technologists	Count	69	8	13		59	18	13	
	% within specialty	76.70%	8.90%	14.40%		65.60%	20.00%	14.40%	
Job grade									
Consultant	Count	85	2	15	0.003	82	5	15	0.007
	% within grade	25.40%	0.60%	4.50%		34.50%	9.40%	34.10%	
Fellow	Count	15	1	4		15	1	4	
	% within grade	4.50%	0.30%	1.20%		6.30%	1.90%	9.10%	
Registrar	Count	80	7	5		67	20	5	
	% within grade	23.90%	2.10%	1.50%		28.20%	37.70%	11.40%	
Resident	Count	15	3	3		13	5	3	
	% within grade	4.50%	0.90%	0.90%		5.50%	9.40%	6.80%	
Radiological technologist/mammographer	Count	67	10	15		57	20	15	
	% within grade	20.00%	3.00%	4.50%		23.90%	37.70%	34.10%	
Other	Count	4	2	2		4	2	2	
	% within grade	1.20%	0.60%	0.60%		1.70%	3.80%	4.50%	
Years of experiences									
1–5 years	Count	88	9	5	0.07	113	48	32	<0.001
	% within Years of experiences	37.30%	3.80%	2.10%		47.50%	90.60%	72.70%	
5–10 years	Count	59	0	1		58	3	10	
	% within Years of experiences	25.00%	0.00%	0.40%		24.40%	5.70%	22.70%	
10–20 years	Count	56	1	2		52	2	2	
	% within Years of experiences.	23.70%	0.40%	0.80%		21.80%	3.80%	4.50%	
More than 20 years	Count	15	0	0		15	0	0	
	% within Years of experiences	6.40%	0.00%	0.00%		6.30%	0.00%	0.00%	

Multivariable binary logistic regression analysis was performed to identify independent predictors of breast density awareness and selected clinical practices after adjusting for age, gender, specialty group, and years of breast imaging experience. Greater years of breast imaging experience was independently associated with higher awareness that breast density affects mammographic accuracy (AOR = 4.48, 95% CI: 2.85**–**7.07, *p* < 0.001) and higher awareness that breast density increases breast cancer risk (AOR = 2.79, 95% CI: 2.04**–**3.80, *p* < 0.001) (Table 5).

**Table 5:** Multivariable logistic regression analysis of independent predictors of breast density awareness.

Outcome	Awareness that BD affects mammographic accuracy		Awareness that BD increases breast cancer risk	
	AOR (95% CI)	*p-*value	AOR (95% CI)	*p*-value
Predictor				
Age	1.01 (0.97–1.04)	0.774	1.00 (0.96–1.03)	0.788
Male vs. Female	0.71 (0.38–1.33)	0.287	0.90 (0.52–1.55)	0.699
Radiologist vs. non-radiology physicians	1.14 (0.53–2.44)	0.742	1.44 (0.75–2.77)	0.277
Radiological technologist vs. non-radiology physicians	1.14 (0.54–2.42)	0.738	1.09 (0.56–2.14)	0.792
Years of breast imaging experience	4.48 (2.85–7.07)	<0.001	2.79 (2.04–3.80)	<0.001

Radiologists were more likely than non-radiology physicians to report routine departmental breast density reporting (AOR = 2.34, 95% CI: 1.28**–**4.30, *p* = 0.006) and routine use of supplementary imaging for women with dense breasts (AOR = 2.98, 95% CI: 1.60**–**5.55, *p* < 0.001). Male participants were less likely to report routinely offering supplementary imaging (AOR = 0.47, 95% CI: 0.27**–**0.81, *p* = 0.007) and less likely to routinely share breast density information with patients (AOR = 0.49, 95% CI: 0.28**–**0.84, *p* = 0.010). Radiological technologists were also less likely than non-radiology physicians to routinely share breast density information with patients (AOR = 0.31, 95% CI: 0.14**–**0.69, *p* = 0.004) (Table 6).

**Table 6:** Multivariable logistic regression analysis of independent predictors of breast density clinical practices.

Outcome	Routine departmental BD reporting		Routine supplementary imaging for increased BD		Routine sharing of BD information with patients	
	AOR (95% CI)	*p*-value	AOR (95% CI)	*p*-value	AOR (95% CI)	*p*-value
Predictor						
Age	0.99 (0.96–1.03)	0.676	1.02 (0.98–1.05)	0.333	0.98 (0.95–1.01)	0.190
Male vs. Female	0.65 (0.38–1.10)	0.106	0.47 (0.27–0.81)	0.007	0.49 (0.28–0.84)	0.010
Radiologist vs. non-radiology physicians	2.34 (1.28–4.30)	0.006	2.98 (1.60–5.55)	<0.001	1.21 (0.66–2.22)	0.545
Radiological technologist vs. non-radiology physicians	1.06 (0.54–2.07)	0.869	1.21 (0.59–2.47)	0.605	0.31 (0.14–0.69)	0.004
Years of breast imaging experience	2.33 (1.80–3.04)	<0.001	2.01 (1.56–2.59)	<0.001	1.26 (0.97–1.64)	0.077

## Discussion

This study assessed the level of knowledge, awareness, and clinical practices related to breast density (BD) among healthcare professionals (HCPs) in Saudi Arabia. Using a cross-sectional design, the study explored how awareness of BD as a factor affecting mammographic sensitivity and breast cancer risk varied according to demographic and professional characteristics. Therefore, it is crucial to investigate and understand how HCPs in Saudi Arabia perceive BD awareness and its implications for breast cancer screening and risk communication.

Overall, the study found relatively high awareness that BD affects mammographic accuracy and is associated with increased breast cancer risk, especially among radiologists, alongside substantial gaps among non-radiology physicians and radiological technologists. Clinical practices such as routine reporting of density and offering supplementary imaging were more common in radiology departments among radiologists, whereas uncertainty was frequent in other specialties. Communication with patients about BD was inconsistent, and many HCPs reported limited confidence and structural barriers (time, lack of guidelines), despite more than half indicating a perceived need for national guidelines on BD management. These findings are particularly important in the Saudi context, where recent work has demonstrated a considerable prevalence of dense breasts and a significant association between BD and breast cancer risk among Saudi women (7, 18, 19), as well as limited public awareness of BD and its implications (5). The observed variation in awareness across professional groups aligns with findings from other Saudi studies. A study conducted at a single Saudi medical center reported that only 41% of healthcare practitioners were well aware of the implications of BD for breast cancer risk (20). Physicians demonstrated higher awareness (37%) compared to nurses and allied health practitioners, with radiologists and surgeons exhibiting the highest levels of understanding (20). Similarly, in the present study, nearly all radiologists were aware that BD affects mammographic accuracy, whereas substantial proportions of non-radiology physicians and radiological technologists either lacked this knowledge or were uncertain. A similar pattern was observed for awareness of the increased breast cancer risk associated with higher BD. When compared with international studies, these findings suggest similar awareness among radiologists in Saudi Arabia but persistent gaps among other HCP groups. In a United States study by Brown et al. (6), 62% of physicians were unaware that dense breasts are associated with increased breast cancer risk, and 67% reported needing more education on BD and supplemental screening. Likewise, a Systematic review among primary care practitioners (PCPs) in the US and a qualitative study among general practitioners (GP) in Australia’s documented low levels of BD knowledge, low comfort in discussing BD with patients, and uncertainty about appropriate management of women with dense breasts and expressed a strong desire for more education and clearer guidance (11, 21).

A notable finding of this study is that approximately two-thirds of participants agreed that HCPs should inform women about the reduced sensitivity of mammography related to BD, and just over half believed women should be informed about the associated increase in breast cancer risk. Despite this, only about a quarter reported routinely sharing BD information with their patients, and one-third did so only when patients specifically asked. Over one-fifth did not share BD information at all. Only 13.1% of HCPs reported that patients ask them about BD, and a relatively small proportion explicitly discussed BD as a cancer risk factor (18.5%), although many advised additional imaging or emphasized breast awareness. These findings are consistent with international evidence highlighting ongoing challenges in BD communication. Nickel et al.’s (11) systematic review found that PCPs often have low comfort in discussing BD, limited consensus on the most appropriate management strategies for women with dense breasts, and a perceived need for clearer guidelines and educational support. Qualitative studies among Australian general practitioners similarly reported concerns about causing unnecessary anxiety, uncertainty in integrating BD into personalized risk discussions, and lack of confidence in recommending supplemental screening (21). In the United Kingdom, an integrative review of BD information sharing screening programs reported that, while women generally want to know their BD status, conversations with HCPs are more effective than letters, but HCPs themselves require better evidence-based resources and training, and potential widening of health inequalities (22).

A particularly important finding in this study is the gap between awareness of BD and routine patient communication. Although awareness that BD affects mammographic accuracy and increases breast cancer risk was relatively high among radiologists, this did not consistently translate into routine discussions with patients. This may reflect a knowledge-practice gap, where healthcare professionals recognize the clinical importance of BD but lack clear pathways for communicating this information in routine practice. It may also reflect uncertainty regarding professional responsibility, as radiologists may focus mainly on imaging interpretation and reporting, whereas referring physicians may be expected to provide direct counseling but may feel less confident discussing imaging-related risk information. In addition, the absence of standardized national guidance on BD notification and management in Saudi Arabia may contribute to inconsistent communication practices.

From an educational perspective, findings presented in this study highlight the need to integrate breast density education into continuing professional development (CPD) programs for healthcare professionals involved in breast cancer screening, diagnosis, and referral pathways. Such programs should not only address factual knowledge of breast density, mammographic masking, and cancer risk, but also include practical training in risk communication, patient counseling, and shared decision-making. Interprofessional education involving radiologists, non-radiology physicians, and radiological technologists may also help clarify professional roles and promote consistent patient communication across the breast care pathway.

Furthermore, more than half of the HCPs in the present study indicated that there is a need for national guidelines on the management of BD in breast cancer screening and diagnosis. This perceived need reflects uncertainty in clinical decision-making, particularly regarding patient counseling, density reporting, and the appropriate use of supplemental imaging for women with dense breasts. Evidence from a recent systematic review underscores the lack of high-quality, evidence-based guidelines to support women and healthcare professionals in navigating complex discussions related to breast cancer screening and BD. The review highlights the need for improved methodological rigor in the development of screening and supplemental imaging guidelines for women with dense breasts, including broader stakeholder engagement. Furthermore, careful consideration is required to ensure that such guidelines are feasible to implement in clinical practice and do not inadvertently exacerbate existing health inequities (23). Given the high prevalence of dense breasts among Saudi women and the growing evidence that dense breasts are associated with both increased risk and reduced mammographic sensitivity, the recognition by Saudi HCPs of the need for national BD guidance is a critical finding for policymakers and professional societies (24).

This study has several limitations that should be considered. The cross-sectional design precludes causal inference, and the use of convenience sampling may have introduced selection bias, as healthcare professionals with greater interest or involvement in breast imaging may have been more likely to participate. Therefore, the findings may overestimate the overall level of breast density awareness among healthcare professionals in Saudi Arabia. In addition, data were collected using a self-administered online questionnaire, which may be subject to self-report and social desirability bias, particularly for questions related to clinical practice and patient communication. Reported practices were not independently verified through medical records, imaging reports, or departmental audits. Furthermore, because many respondents were likely from hospitals, breast-imaging centers, oncology services, and other specialized or tertiary healthcare settings, the findings may not be fully generalizable to all healthcare professionals working in primary care, rural areas, or non-tertiary institutions across Saudi Arabia. One additional limitation is that the final sample size was lower than the targeted sample size. Although the minimum required sample size was calculated as 385 participants, the final sample included 335 participants due to a lower-than-expected response rate. This shortfall was considered when interpreting the findings, as it may have reduced the statistical power of the study. Moreover, because the questionnaire was distributed through the SCHS online platform, data on the exact number of invitations sent, delivery rates, and open rates were not available; therefore, a formal response rate could not be calculated, which limits the ability to assess potential non-response bias. Despite these limitations, the study provides valuable insight into current knowledge gaps and practice variations related to breast density awareness and communication among healthcare professionals.

## Conclusion

This cross-sectional study evaluated healthcare professionals’ knowledge, awareness, and practices related to BD in Saudi Arabia. The findings demonstrate that awareness of the impact of BD on mammographic sensitivity and breast cancer risk was higher among radiologists than among non-radiology physicians and radiological technologists, among whom substantial knowledge gaps and uncertainty were observed. Routine reporting of BD and the use of supplementary imaging were more commonly practiced within radiology departments by radiologists, whereas many participants outside radiology were unsure whether such practices were consistently implemented. Although most respondents agreed that women should be informed about BD and its clinical implications, patient communication was inconsistent, and only a minority routinely discussed BD as a breast cancer risk factor or provided structured counseling. Collectively, these findings underscore the need for targeted continuing professional development, interprofessional educational interventions, standardized breast density knowledge dissemination, and context-specific national guidance to support risk-stratified screening and strengthen early breast cancer detection efforts in Saudi Arabia.

## Data Availability

The original contributions presented in the study are included in the article/Supplementary material, further inquiries can be directed to the corresponding author.
