# Supplementary material for: Assessment of knowledge and awareness regarding breast density among breast cancer screening healthcare professionals
Source: Front Med (Lausanne). 2026 Jul 9;13:1843814. doi: 10.3389/fmed.2026.1843814 (PMC13391512; doi:10.3389/fmed.2026.1843814)
Supplement: Supplementary file 1 [file Data_Sheet_1.docx]

**Supplementary File 1. Breast Density Knowledge and Awareness Questionnaire**

Dear [Healthcare Professional],

We are inviting you to participate in a survey for a study that assesses healthcare professionals' knowledge and attitude regarding breast density. This study aims to identify the level of understanding and perception of healthcare professionals in Saudi Arabia towards breast density, which is a significant factor in breast cancer screening.

We value your expertise and experience in the healthcare field, and we believe that your participation in this study will contribute significantly to the research's success. The survey will take approximately 5-10 minutes to complete.

Your participation is entirely voluntary, and you are free to withdraw at any time during the survey. We assure you that all data collected will be kept confidential and only used for research purposes.

Thank you in advance for considering our invitation. Your contribution to this study is highly appreciated. If you have any questions or concerns regarding the survey or the study, please do not hesitate to contact me at 0118050471 or via email: aaloufi@ksu.edu.sa.

Do you agree to participate?

- Yes
- No

Sincerely,

Areej Aloufi BSc, MSc, PhD

**Breast Density Knowledge and Awareness Questionnaire**

1. **Age:**

*Short answer*

1. **Gender:**

- Male
- Female

1. **Job title:**

*Short answer*

1. **What grade are you?**

*Mark only one oval.*

- Consultant
- Registrar
- Resident
- Fellow
- Radiological technologist
- Other:

1. **What type of breast imaging experience do you have? Tick all that apply**

- Breast Ultrasound
- Mammography
- Breast MRI
- Digital Breast Tomosynthesis (DBT)
- Contrast-Enhanced Mammography
- None

1. **How many years of experience do you have in breast imaging?**

- 1–5 years (or less)
- 5–10 years
- 10–20 years
- >20 years
- None

1. **Are you aware that mammographic accuracy is affected by breast density?**

- Yes
- No
- Unknown

1. **Are you aware of the relative risk for breast cancer by the degree of breast density?**

- Yes
- No
- Unknown

1. **Does your department routinely report on mammographic density?**

- Yes
- No
- I don’t know

1. **If your department uses ACR BI-RADS breast density categories, which category(s) are considered to be “dense breasts?”**

- Category A
  - Category B
  - Category C
  - Category D
  - Category C & D
  - Not applicable

1. **Do you routinely offer supplementary imaging for women with increased breast density?**

- Yes
- No
- Unknown

1. **Are you concerned that routine supplementary imaging could result in over-investigation of false positive findings?**

- Yes
- No
- I don’t know

1. **Should healthcare providers (such as radiologists or primary physicians) inform women of the reduced sensitivity of mammograms in the context of dense breasts?**

- Yes
- No
- I don’t know

1. **Should healthcare providers (such as radiologists or primary physicians) inform women of the increased breast cancer risk in the context of dense breasts?**

- Yes
- No
- I don’t know

1. **Do your patients ask about breast density?**

- Yes
- No
- I don’t know

1. **Do you share breast density with your patients?**

- Yes
- No
- Only if asked
- None

1. **What advice regarding breast density do you offer your patients? Tick all that apply.**

- Breast awareness and breast examination
- Increased breast density may increase breast cancer risk
- Increased breast density may reduce the accuracy of a mammogram.
- You may benefit from additional imaging due to your breast density

1. **Why don’t you share breast density information with your patients? Tick all that apply.**

- Do not have this information available
- Do not feel this information should be shared if no alternative imaging is offered
- Do not feel confident sharing this information.
- Time restraints
- Other:

1. **Do you think there is a need for further guidelines in Saudi Arabia on the management of breast density?**

- Yes
- No
- None
